# Therapeutic effects by the peptide drug of p3‐Alcß in AD mouse model

**DOI:** 10.1002/alz70859_101763

**Published:** 2025-12-25

**Authors:** Toshiharu Suzuki, Haruka Saito, Shoichi Kinoshita, Kanae Ando, Saori Hata

**Affiliations:** ^1^ Tokyo Metropolitan University, Hachioji Japan; ^2^ Hokkaido University, Sapporo Japan; ^3^ Tokyo Metropolitan University, Hachioji, Tokyo Japan; ^4^ National Institute of Advanced Industrial Science and Technology (AIST), Sapporo Japan

## Abstract

**Background:**

Alcadeinß (Clstn3), a synaptic membrane protein, is cleaved by α‐ and γ‐secretases, which secrets 37 and 40 amino acids non‐aggregable peptide p3‐Alcß37 and p3‐Alcß40 (*J. Biol. Chem*. 284: 36024‐36033 [2009]). The peptides and the shorter 11 amino acids peptide of p3‐Alcß (p3‐Alcß9‐19) show an ability to restore mitochondria activities that are damaged in neurons by Aß oligomers and in brains of AD mouse model (*EMBO Mol. Med*. 15: e17052 [2023]). Here we tried to identify molecular targets and their validation by analyzing gene expression profiles in brain regions of AD mouse model administrated with p3‐Alcß9‐19.

**Method:**

*App^NL‐F/NLF^* mice (*Nat. Neurosci*. 17:661‐663 [2014]) were subcutaneously administrated with p3‐Alcß9‐19 (1mg/kg) for a month (twice in a week). The brain regions were dissected, and the extracted RNA was subject to RNAseq analysis. Gene expression levels were compared with the results of RNAseq analysis of mice subjected with PBS. The relationship between mouse genes showed expressional changes by p3‐Alcß9‐19 administration and human genes that changed the expression in AD were also analyzed.

**Result:**

Administration of p3‐Alcß9‐19 in AD mouse model changed the expression of genes that are not only neuron but also non‐neuronal cells. Many of mouse ortholog genes that downregulated in human AD patients were upregulated or tended to upregulate in AD mouse model which were administrated with p3‐Alcß not PBS. On the contrary, some ortholog of genes that upregulated in human AD patients were revised in AD mouse model by p3‐Alcß9‐19 administration.

**Conclusion:**

Results suggests that p3‐Alcß protects neuronal functions from impairments of brain by revising gene expression profiles which altered by the accumulation of pathogenic and/or denatured proteins in brains. Therefore, the p3‐Alcß may be effective as a drug to preserve brain homeostasis from neurodegeneration. Our preclinical observations should light on the clinical trial with the ALC919, the transdermal drug formular of p3‐Alcß9‐19.

